# Hyperphosphatemia is associated with anemia in adults without chronic kidney disease: results from the National Health and Nutrition Examination Survey (NHANES): 2005–2010

**DOI:** 10.1186/1471-2369-14-178

**Published:** 2013-08-21

**Authors:** Janet M Wojcicki

**Affiliations:** 1 Department of Pediatrics, University of California, San Francisco, CA 94134-0136, USA

**Keywords:** Hyperphosphatemia, Hypophosphatemia, Lower socioeconomic status, Anemia, Obesity

## Abstract

**Background:**

Hyperphosphatemia, serum phosphorus ≥ 4.4 mg/dL, is associated with increased risk for chronic kidney disease and cardiovascular disease. Previous studies have shown a weak association between dietary phosphorus intake and serum phosphorus concentrations. While much less common in the general population, hypophosphatemia (< 2.5 mg/dL) may be associated with metabolic syndrome and obesity.

**Methods:**

Using three cycles from the National Health and Nutrition Examination Survey (NHANES) (2005–2010), this study evaluated independent risk factors for hyperphosphatemia and hypophosphatemia.

**Results:**

Risk factors for hyperphosphatemia included higher adjusted calcium (OR 2.90, 95% CI 2.43-3.45), increasing cholesterol (OR 1.003, 95% CI 1.001-1.005), female gender (OR 1.61, 95% CI 1.39-1.87) and low hemoglobin (OR 1.52, 95% CI 1.17-1.98). Advanced age was protective (OR 0.98, 95% CI 0.977-0.987). Models that included fasting serum glucose found lower body mass index (BMI) to be protective (OR 0.97, 95% CI 0.96-0.99) and adjusting for serum vitamin D and parathyroid hormone removed the association with low hemoglobin and BMI. Risk factors for hypophosphatemia included the following protective factors: higher albumin (OR 0.56, 95% CI 0.35-0.93), higher BUN (OR 0.90, 95% CI 0.86, 0.95), corrected calcium (OR 0.38, 95% CI 0.23-0.63) and female gender (OR 0.47, 95% 0.24-0.94). In men, higher fasting glucose levels increased risk (OR 1.01, 95% CI 1.0004-1.01).

**Conclusion:**

This study is the first to show an association between low hemoglobin levels and increased risk for hyperphosphatemia among individuals without chronic kidney disease. We did not find any association between diabetes mellitus, increasing BMI or fasting glucose levels and hypophosphatemia.

## Background

Determinants of serum phosphorus concentrations include dietary intake, gastrointestinal absorption of phosphorus, urinary excretion and shifts in phosphorus between the intracellular and extracellular spaces [1]. Previous studies have found only a weak association between serum phosphate concentrations and dietary phosphorus intake [2]. There has been much focus on trying to better understand the risk factors for hyperphosphatemia, as elevated phosphate levels are associated with progress of chronic kidney disease (CKD) and end stage renal disease (ESRD) [3]. Even levels in high normal range (≥ 4 mg/dL & < 4.5 mg/dL) have been found to be associated with increased risk for chronic kidney disease [3]. Elevated serum phosphorus is also associated with cardiovascular disease risk through the potential mechanism of vascular calcification via upregulation of core binding factor a-1 in vascular smooth muscle cells in both those with and without CKD [4].

### Risk factors for hyperphosphatemia: poverty and environment

A recent study evaluating risk factors for hyperphosphatemia found an association between elevated phosphate levels and poverty, although it is not clear which intermediary factors associated with poverty would simultaneously increase phosphate levels [2]. It is possible that increased environmental exposures to pesticides and phenols may mediate the relationship between the association found between low socioeconomic status and hyperphosphatemia. Previous studies found that urinary excretion of environmental phenols (specifically bisphenol A (BPA) and triclosan) were lower in individuals with reduced estimated glomerular filtration rate (eGFR) although it is not clear if they could similarly be associated with increased serum phosphorus levels [5]. Moreover, these relationships have not been investigated in adults with normal eGFR.

### Anemia and hyperphosphatemia in ESRD

In patients with ESRD and kidney transplant recipients, a previous study [6] found a relationship between hyperphosphatemia and anemia. Various components of CKD mineral and bone disorders (CKD-MBD) may result in a direct suppressive effect on erythropoiesis, resulting in anemia, including low vitamin D, calcium and increased serum parathyroid hormone levels. It is not clear whether the relationship between hyperphosphatemia and anemia is mediated by other aspects of CKD-MBD in CKD patients. A study of the relationship between kidney transplant recipients and high-normal phosphate levels found a significant association of higher serum phosphorus level with lower hemoglobin, independent of other factors associated with CKD-MBD and known determinants of anemia [6]. Kovesdy et al., [6] theorize that the relationship between phosphorus and anemia may be mediated by uremic polyamine metabolism. Polyamines are uremic toxins that inhibit erythropoiesis [7]. Higher levels of polyamines as a result of phosphorus levels could explain the relationship between higher phosphorus levels and anemia. Meanwhile, although these studies suggest an important relationship between hyperphosphatemia and anemia in specific types of patients, there are no studies evaluating the relationship between hyperphosphatemia and anemia in patients without kidney disease.

### Female gender and hyperphosphatemia

Other known risk factors for hyperphosphatemia include female gender although the reasons for this association are unclear [8]. One study suggests that estrogen may act directly to suppress sodium-dependent phosphate absorption in the renal proximal tubules inducing phosphaturia and decreased serum phosphate; women who are post-menopausal and estrogen deficient would be at increased risk for hyperphosphatemia [9].

### Risk factors for hypophosphatemia

In contrast with hyperphosphatemia, hypophosphatemia is much less common in the general, non-hospitalized population. Low serum phosphate levels are associated with reduced cardiac output and also with risk for arrhythmia [10]. However, recent studies have suggested that hypophosphatemia is more common than previously recognized as there may be an association between obesity and the metabolic syndrome and risk for hypophosphatemia [11]. Haglin theorizes that a low serum phosphate level limits phosphorylation of carbohydrate intermediates in glycolysis and glycogenesis and chronic hypophosphatemia inhibits glucose transport potentially resulting in diabetes mellitus and hypertension [11].

The aim of the present study was to evaluate potential risk factors for hyperphosphatemia and hypophosphatemia in a large population based survey of patients without kidney disease, the National Health and Nutrition Examination Surveys (NHANES) from 2005–10, including anemia as well as investigate the possible relationship between exposure to environmental phenols, specifically and mediating the association between poverty and hyperphosphatemia.

## Methods

### Study population

The NHANES is a nationally representative, cross-sectional survey, which was designed by the National Center of Health Statistics (NCHS) to be a representative sample of the non-institutionalized US population and used to assess the health and nutritional status of Americans. A total of 31,034 adults and children participated in NHANES 2005–2010 (three cycles 2005–6; 2007–2008; 2009–10). These NHANES cycles were approved by the Institutional Review Board of the National Center for Health Statistics. All NHANES participants who were ≥18 years of age provided informed consent.

**Figure 1:**
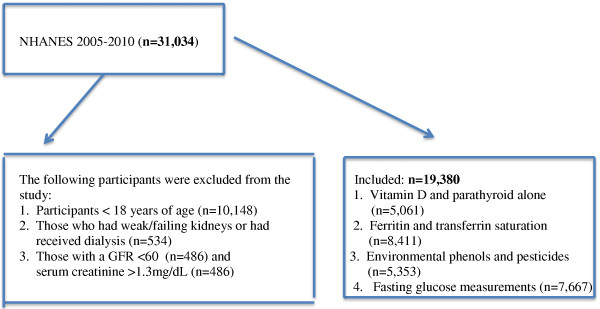
Inclusion and exclusion of NHANES 2005-2010 participants.

Participants <18 years of age were excluded from this study (n =10,148). Participants who stated that they had been told that they had weak/failing kidneys or had received dialysis in the past 12 months (n = 534) were also excluded from the study as were those with an estimated glomerular filtration rate <60 (n=486) and a serum creatinine > 1.3 mg/dL (n = 486). Vitamin D and parathyroid measurements were only available from the 2005–6 cycle (n = 5061) and ferritin and transferrin receptor saturation were only available on women for 2005–2010 for a smaller sample size (n = 8,411). For the environmental phenols and pesticides, only one third of the sample was used per cycle (approximately 2,500 participants depending on the years surveyed without the above exclusions) and special sampling weights provided by NHANES were used for each cycle. For the fasting glucose measurements, only those participants who were examined in the morning sample were included in sample (approximately 3,000 per cycle without the above exclusions) (Figure 1). As described below in results, the exact number of participants varied for different analyses that were conducted.

### Study variables

For the laboratory measurement, different methods were used in the first cycle (2005–6) versus subsequent cycles (2007–8; 2009–10).

#### Biochemistry

In NHANES 2005–6, serum creatinine, blood urea nitrogen (BUN), calcium, total cholesterol, phosphorus, uric acid and albumin were measured using the Beckman Synchron LX20 method (Brea, CA) [12]. Similarly, total calcium and uric acid were measured with a Beckman Synchron analyzer (Brea, CA). Different laboratory methods were used for calculating creatinine as described below depending on year of NHANES survey; however, all of these were standardized so that they could be combined for analyses. Creatinine was calculated in 2005–6, 2007–8, 2009–10 using the Jaffe rate method (kinetic alkaline picrate) to determine the concentration of creatinine in serum where the creatinine calibration was traceable to an isotope dilution mass spectrometry (IDMS) reference method [12-14]. In 2008 and 2009–10, the Beckman DxC800 was used. In 2005–6 and 2007, the Beckman LX-20 was used, however, a Deming regression was applied to standardize creatinine values between 2005–6 and 2007 and 2008, and 2009–10. In these years when the LX-20 was used, standard creatinine values (mg/dL) were corrected using the following equation: -0.016+0978 * (NHANES 05–06 uncalibrated serum creatinine, mg/dL) [12,13]. In 2007–2010, albumin, calcium, total cholesterol, phosphorus, uric acid and blood urea nitrogen (BUN) were measured using the DxC800 method [13,14]. Corrected calcium values were calculated for participants with albumin levels <3.4 g/dL. In 2009–10, Glycohemoglobin measurements were performed on the A1c 2.2 Plus Glycohemoglobin Analyzer (Toshoh Medics, Inc., 347 Oyster Pt. Blvd., Suite 201, So. San Francisco) [15]. For the early cycles (2005–8), glycohemoglobin measurements were performed on the A1c G7 HPLC Glycohemoglobin Analyzer (Tosho Medics, Inc., 347 Oyster Pt. Blvd., Suite 201, So. San Francisco) [16,17]. C-reactive protein was quantified by latex-enhanced nephelometry in all years. Particle-enhanced assays were based on the reaction between a soluble analyte and the corresponding antigen or antibody bound to polystyrene particles. For the quantification of CRP, particles consisting of a polystyrene core and a hydrophilic shell were used to link anti-CRP antibodies covalently [18-20]. Ferritin measurements were conducted using the Roche/Hitachi 912 clinical analyzer in all years [21-23] and soluble transferrin receptor was measured with immune-turbidimetry using Roche kits on the Hitachi 912 clinical analyzer [24-26]. Fasting glucose concentration was determined by a hexokinase method in all years [27-29] and the fasting blood test was performed after a 9 hour fast. In 2005–6, parathyroid hormone levels were evaluated using the Elecysys 1010 analyzer using the ECL/Origen electrochemiluminescent process [30] and 25-hydroxyvitamin D (25-OH-Vitamin D) was assessed by the Diasorin assay using a two-step procedure [31].

#### Environmental data

The environmental phenols (bisphenol A (BPA), benzopheonone-3 (BP-3) and one cholorphenols triclosan were measured in all years using solid phase extraction coupled on-line to high performance liquid chromatography [32-34].

#### 24-hour dietary recalls

Two 24 -hour dietary recalls were used to assess the intake of specific micronutrients including phosphorus. In order to ensure accuracy, the dietary recall interview included a set of 3 dimensional measuring guides and the interview asked if the dietary intake was usual or unusual for that person and then the material is provided as an attachment as described in the NHANES documentation [35]. Other measures are also in place, including periodically audiotaping the interviews to ensure that methods are being appropriately followed [35]. The mean intake of phosphorus was subsequently calculated averaging over two days.

#### Calculations and definitions

For all years, the estimated glomerular filtration rate was then calculated using the Modification of Diet in Renal Disease Study equation [36], which is eGFR (mL/min/m [2]) =175.0×(Scr^)-1.154^ × age^-0.203^ × 0.742 (if female) × 1.212 (if black). Furthermore, the CKD-Epi formula was also used to estimate GFR where eGFR=144 × min (SCr/k,1)^a^ × max (SCr/k,1)^-1.209^ × 0.993^Age^ [1.018 if Female] × [1.159 if Black] [37]. Low hemoglobin was defined as <12 g/dL for women and <13 g/dL for men. Low hematocrit was defined as < 36% for women and < 41% for men. Low ferritin was defined as < 12 ng/mL for women and men and high transferrin receptor saturation defined as > 4.6 g/L.

Demographic information was also collected by NHANES including information on the participants’ gender, age, race/ethnicity with race/ethnicity defined as non-Hispanic white, non-Hispanic black, Mexican-American, other Hispanic and other race. Body mass index was calculated based on weight and height measurements with weight (kilograms) divided by height (meters squared). Diabetes mellitus was defined as a self-reported physician diagnosis of diabetes at the time of the interview. Poverty to income ratio was used as the main variable to assess poverty status, which is an index calculated by dividing family income by a poverty threshold specific to family size. Living in poverty was defined as having a poverty-to income ratio < 1. Obesity was defined as having a body mass index (BMI) ≥ 30 kg/m [2]. Diabetes mellitus, kidney disease (“failing kidneys”) and dialysis use was defined through self-report, with information collected via questionnaire format.

Steroid use was categorized as yes if any participant reported the use of prednisone, prednisolone, methylprednisolone, or methylprednisolone acetate within the last 30 days. This data was extracted from the medication questionnaire.

### Statistical analysis

Serum phosphate levels were dichotomized by presence (≥ 4.4 mg/dL) or absence (< 4.4 mg/dL & ≥ 2.5 mg/dL) of hyperphosphatemia or presence (< 2.5 mg/dL) or absence (≥ 2.5 mg/dL & <4.4 mg/dL) of hypophosphatemia. Additionally hyperphosphatemia was also evaluated in relationship to lower levels of serum phosphate (< 3.5 mg/dL). Bivariate analyses were conducted for risk factors for hyperphosphatemia or hypophosphatemia and included an evaluation of biochemical, sociodemographic, pesticide and phenol exposure and health history and dietary variables. Variables that were significant at p < 0.05 were included in multivariate logistic regression models analyzing independent risk factors for hyperphosphatemia or hypophosphatemia. All analyses were conducted using Stata 12.0.

Sample weights were used as provided by NHANES in the analysis to combine weights from all three cycles following the NHANES analytic guidelines [38]. As described above, special weight were used for the phenol and pesticide samples and also for any analysis that included fasting glucose levels. As vitamin D and parathyroid hormone levels were only available for the 2005–6 cycle, all analysis that included either of these variables used the sampling weights from the 2005–6 cycle alone. The study was exempted from approval by the Committee on Human Research (CHR) at the University of California, San Francisco (UCSF).

## Results

There were 19,380 persons ≥18 years of age from the three cycles of the NHANES (2005–2010) that were included in the analysis after exclusions based on age, laboratory parameters or self-reported conditions, although a smaller number was available in multivariate analyses based on missing data for certain parameters. A smaller number from the 2005–6 cycle included data on vitamin D and parathyroid hormone (n = 5,061), as well as those that had morning blood draw for fasting glucose (n= 7,667) and were assessed for environmental phenol exposures (n = 5,353). One hundred and forty-four reported current use of oral steroids > 30 days, which represented a weighted percent of 0.50% (95% CI 0.41-0.61). In the total population evaluated, 15.29% (95% CI 14.23-16.41) had hyperphosphatemia (phosphorus ≥ 4.4 mg/dL) and 0.59% (95% CI 0.43-0.82) had hypophosphatemia (phosphorus ≤ 2.5 mg/dL).

### Risk factors for hyperphosphatemia

In bivariate analysis, risk factors for hyperphosphatemia included higher levels of albumin (p < 0.01), higher vitamin D (p = 0.01), lower parathyroid hormone (p = 0.02), higher corrected calcium (< 0.01), lower fasting glucose (< 0.01) and lower hemoglobin levels (p = 0.03) (Table 1). Low hemoglobin levels were associated with increased risk for hyperphosphatemia with 6.62% of those having a phosphate ≥ 4.4 having low hemoglobin in contrast with 5.04% of those having phosphate less than 4.4 but > 2.5 (p = 0.03) (Table 1). Women were more likely to have high serum phosphate (17.66% versus 12.73%, p < 0.01) as were younger participants (42.34±0.49 versus 45.91±0.65 years, p < 0.01). Lower body mass index (BMI) was associated with increased risk for hyperphosphatemia (27.86±0.20 kg/m [2] versus 28.75±0.25 kg/m [2]; p < 0.01).

**Table 1:** Association between hyperphosphatemia and selected biochemical, socio-demographic and health history variables

	**Phosphorus ≥ 4.4 mg/dL**	**Phosphorus < 4.4 mg/dL**	**P value**
	**Means±SE or %**	**Means±SE or %**	
Biochemical			
Albumin (g/dL)	4.31±0.01	4.26±0.01	<0.01
Blood urea nitrogen (BUN) (mg/dL)	12.80±0.16	12.66±0.40	0.74
BUN ≥ 20 mg/dL	6.78	8.63	0.51
Vitamin D (25-0H-D) (ng/ml)	24.45±0.51	22.81±0.60	0.01
Vitamin D <10 (25-OH-D) (ng/ml)	4.05	5.58	0.12
Vitamin D >30 (25-OH-D) (ng/ml)	21.82	16.68	0.10
Parathyroid hormone(pg/mL)	38.25±1.48	44.09±1.64	0.02
Total calcium (mg/dL)	9.57±0.01	9.44±0.01	<0.01
Corrected calcium (mg/dL)	9.59±0.01	9.44±0.01	<0.01
Calcium >10 mg/dL	7.19	3.99	<0.01
Cholesterol (mg/dL)	202.14±1.15	194.90±1.88	<0.01
Serum creatinine (mg/dL)	0.84±0.004	0.86±0.01	0.11
Uric acid (mg/dL)	5.29±0.04	5.45±0.08	0.10
eGFR (MDRD)	94.57±1.01	92.31±0.73	0.051
eGFR (CKD-Epi)	98.16±0.60	95.90±1.09	0.052
Hemoglobin A1c	5.49±0.02	5.54±0.03	0.15
Fasting glucose (mg/dL)	99.52±0.86	103.99±0.52	<0.01
C-reactive protein (mg/dL)	0.36±0.02	0.37±0.01	0.55
Anemia and Iron			
Low hemoglobin levels^*^	6.62	5.04	0.03
Low hematocrit levels^	13.2	11.44	0.07
Low hemoglobin or hematocrit	13.51	11.69	0.08
Low Ferritin <12 ng/mL	12.78	11.96	0.60
High Transferrin Sat > 4.6 g/L	16.02	14.36	0.36
Socio-demographics			
Gender			
Male	12.73	87.27	<0.01
Female	17.66	82.34	
Age (years)	42.34±0.49	45.91±0.65	<0.01
Ethnicity			
Mexican	14.16	85.84	0.56
Other Hispanic	14.24	85.76	
White	15.29	84.71	
Black	15.16	84.84	
Other Race	16.65	83.35	
Poverty income Ratio	3.07±0.06	3.08±0.05	0.90
Poverty income Ratio <1	14.54	13.07	0.14
Health History and Diet			
Body Mass Index (kg/m^2^)	27.86±0.20	28.75±0.25	<0.01
Obesity (BMI ≥ 30 kg/m2)	31.32	36.08	0.08
Self reported diabetes			
Yes	7.76	92.24	0.07
No	16.32	83.68	
Phosphorus Intake mg/day	1410.25±21.91	1364.69±23.43	0.14
Steroids (>past 30 days, any)	0.70	0.44	0.20
Envrionmental Phenols			
Urinary Benzopheone-3 (ng/mL)			
≤6	15.62	84.38	0.13
>6 & ≤30.1	13.07	86.93	
>30.1	16.98	83.02	
Urinary Bisphenol A (ng/mL)			
<1.3	16.05	83.95	0.72
≥1.3 & <3	14.45	85.55	
≥3	15.47	84.53	
Urinary Tricolsan (ng/mL)			
≤4.2	15.78	84.22	0.63
>4.2 & <23.3	16.07	83.93	
≥23.3	14.51	85.49	

^*^Low hemoglogin is defined as <12g/dL for women and <13g/dL for men.

^ Low hematocrit was defined as <36% for women and <41% for men.

In examining the association between urinary pesticide and phenol exposures, there was no association between levels of bisphenol A (BPA), benzopheone-3 (BP-3) and tricolsan and hyperphosphatemia. We also evaluated risk factors for high serum phosphate ≥ 4.4 in comparison to those with low serum phosphate < 3.5 finding similar risk factors to those described above with the exception of a higher BUN and a lower uric acid and serum creatinine in those with hyperphosphatemia in comparison with those with a serum phosphate < 3.5. In multivariate logistic regression analysis (n=15,578), increasing calcium (OR 2.90, 95% CI 2.43-3.45), higher cholesterol (OR 1.003, 95% CI 1.001-1.005), female gender (OR 1.61,95% CI 1.39-1.87) and low hemoglobin (OR 1.52, 95% CI 1.17-1.98) were associated with increased risk for hyperphosphatemia (Table 2). Older age was protective against hyperphoshatemia (OR 0.98, 95% CI 0.977-0.987) as was lower GFR (OR 0.996, 95% CI 0.992-0.999) (Table 2). Adjusting for serum glucose levels using a smaller sample size (n=7,631), independent risk factors for hyperphosphatemia included corrected calcium (OR 3.59, 95% CI 2.55-5.05), female gender (OR 1.83, 95% CI 1.51-2.23) and low hemoglobin (OR 1.52, 95% CI 1.003-2.29). When the model used a smaller sample size (n=5,019) but included vitamin D and parathyroid hormone levels, lower BMI was no longer protective and low hemoglobin no longer increased risk (Table 2). Similar risk factors were present for hyperphosphatemia when compared with serum phosphate levels < 3.5 in multivariate analysis although increasing serum BUN was also associated with hyperphosphatemia. In both the larger model and the model with fasting glucose, low hemoglobin was associated with increased risk (OR 1.43, 95% CI 1.09-1.86 for large model and OR 1.60, (95% CI 1.07-2.40) for the model with fasting glucose (Table 3)). In a multivariate model with serum vitamin D and parathyroid hormone, increasing parathyroid hormone was associated with decreased risk (OR 0.99, 95% CI 0.98-0.9996) (Table 3).

**Table 2:** Multivariate logistic regression analysis for risk factors for phosphorus ≥ 4.4

**Variables**		** OR 95% CI**	
	**Model 1 (n =15,578)**	**Model 2 (n =7,631)**	**Model 3 (n = 5,019)**
Albumin (g/dL)	1.14 (0.92-1.41)	0.68 (0.50-0.94)^b^	1.22 (0.96-1.55)
Corr. Calcium (mg/dL)	2.90 (2.43-3.45)^a^	3.59 (2.55-5.05)^a^	2.95 (2.14-4.05)^a^
Cholesterol (mg/dL)	1.003 (1.001-1.005)^a^	1.0003 (0.998-1.003)	1.004 (1.00-1.01)^b^
Gender (Female)	1.61(1.39-1.87)^a^	1.83 (1.51-2.23)^a^	1.66 (1.25-2.17)^b^
Age, years	0.98(0.977-0.987)^a^	0.98 (0.97-0.99)^a^	0.98 (0.971-0.99)^a^
BMI (kg/m^2^)	0.99 (0.98-1.005)	0.97 (0.95-0.99)^b^	0.99 (0.96-1.02)
Low Hemoglobin	1.52 (1.17-1.98)^a^	1.52 (1.003-2.29)^b^	1.48 (0.87-2.51)
GFR (CKD-Epi)	0.996 (0.992-0.999)^b^	0.996 (0.989-1.003)	0.99 (0.989-1.00)
Fasting Glucose (mg/dL)		0.999 (0.995-1.002)	
Vitamin D 25-OH-D (ng/ml)			1.00 (0.99-1.01)
Serum PTH (pg/ml)			0.99 (0.98-1.00)

^a^p<0.01.

^b^p<0.05.

**Table 3:** Multivariate risk factors for phosphorus ≥ 4.4 mg/dL in comparison with phosphorus <3.5 mg/dL) (n=12335)

**Variable**		**Odds Ratio (OR) 95% CI**	
	**Model 1 (n=12236)**	** Model 2 (n=6351)**	**Model 3 (n=3752))**
Albumin (g/dL)	1.16 (0.92-1.45)	0.65 (0.46-0.90)^b^	1.24 (0.98-1.59)
BUN (mg/dL)	1.08 (1.06-1.09)^a^	1.07 (1.04-1.10)^a^	1.08 (1.05-1.12)^a^
Corr. Calcium (mg/dL)	3.42 (2.82-4.15)^a^	4.32 (3.15-6.00)^a^	3.61 (2.45-5.93)^a^
Cholesterol (mg/dL)	1.003 (1.002-1.005)^a^	1.0005 (1.00-1.003)^b^	1.00 (1.00-1.01)^b^
Uric Acid (mg/dL)	0.99 (0.94-1.05)	1.11 (1.01-1.22)^b^	1.00 (0.89-1.13)
Gender (Female)	1.92 (1.65-2.24)^a^	2.48 (1.94-3.16)^a^	1.95 (1.45-2.62)^a^
Age (Years)	0.98 (0.976-0.98)^a^	0.97 (0.967-0.98)^a^	0.98 (0.97-0.99)^a^
BMI (kg/m^2^)	0.99 (0.98-1.004)	0.96 (0.94-0.98)^a^	0.99 (0.96-1.02)
Low Hemoglobin	1.43(1.09-1.86)^a^	1.60 (1.07-2.40)^b^	1.28 (0.68-2.40)
Creatinine (ng/mL)	0.98 (0.65-1.48)	0.59 (0.24-1.47)	1.13 (0.55-2.30)
Fasting Glucose (mg/dL)		1.00 (0.995-1.002)	
Vitamin D- 25-OH-D (ng/mL)			0.997 (0.99-1.007)
Parathyroid Hormone (pg/mL)			0.99 (0.98-0.9996)^b^

^a^p<0.01.

^b^p<0.05.

### Hypophosphatemia

Risk factors for hypophosphatemia included a lower albumin (p < 0.01), lower BUN (p=0.02), higher corrected calcium level (p < 0.01) and higher uric acid (p = 0.02). Lower ferritin was protective (p < 0.01), as was being female (p = 0.01) and having a lower BMI (p < 0.01). Being obese increased risk (p = 0.06) (Table 4). In multivariate analysis for independent risk factors for hypophosphatemia included the following factors: albumin and BUN were protective (OR 0.56, 95% CI 0.35-0.93 and OR 0.90, 95% CI 0.86-0.95, respectively) as was higher corrected calcium (OR 0.38, 95% CI 0.23-0.63) and being female (OR 0.47, 95% CI 0.24-0.94) (Table 5). With low ferritin (<12 g/L) in the model, increased creatinine was significant (OR 23.48, 95% CI 1.10-501.47) and low ferritin was protective (OR 0.14, 95% CI 0.04-0.45). Including parathyroid hormone into the model, gender was no longer significant but higher BUN continued to be protective (OR 0.83, 95% CI 0.76-0.91) as did corrected calcium (OR 0.22, 95% CI 0.07-0.73) and higher uric acid was associated with increased risk (OR 1.35, 95% 1.09-1.66). Stratifying based on gender, males were at increased risk for hypophosphatemia with increasing glucose levels (OR 1.01, 95% CI 1.0004-1011); this association was not present in females.

**Table 4:** Association between hypophosphatemia and selected biochemical, socio-demographic and health history variables

	**Phosphorus >2.5 mg/dL**	**Phosphorus ≤ 2.5 mg/dL**	**P Value**
**Variable**	**Means±SE or %**	**Means±SE or %**	
Biochemical			
Albumin (g/dL)	4.18±0.02	4.26±0.007	<0.01
BUN (mg/dL)	12.69±0.41	11.48±0.30	0.02
BUN≥20 (mg/dL)	2.26	8.46	0.02
Vitamin D (25-0H-D) (ng/mL)	21.10±1.34	22.82±0.60	0.13
Vitamin D (25-OH-D) <10 ng/mL	11.16	5.60	0.14
Vitamin D (25-OH-D) >30 ng/mL	12.21	16.73	0.44
Corrected calcium (mg/dL)	9.44±0.01	9.33±0.03	<0.01
Calcium>10 (mg/dL)	4.32	3.99	0.82
Parathyroid pg/mL	44.02±1.65	54.09+±4.74	0.07
Cholesterol (mg/dL)	194.95±1.91	191.37±3.24	0.35
Creatinine (mg/dL)	0.86±0.01	0.89±0.01	0.10
Uric Acid (mg/dL)	5.45±0.0.08	5.71±0.10	0.02
Hemoglobin A1c	5.62±0.10	5.54±0.03	0.42
Fasting glucose (g/mL)	109.26±3.83	103.88±0.52	0.17
eGFR(MDRD)	92.35±0.73	91.58±1.57	0.69
eGFR (CKD-Epi)	96.25±0.96	95.88±1.83	0.86
C-Reactive Protein (mg/dL)	0.37±0.01	0.61±.16	0.15
Anemia and Iron			
Low Hemoglobin^*^	4.18	5.06	0.59
Low Hematocrit^	1.03	1.14	0.63
Low Hemoglobin	1.05	1.17	0.67
Or Hematocrit			
High Trans Sat>4.6 ng/dL	12.27	14.35	0.71
Low Ferritin <12 g/L	1.85	12.2	<0.01
Sociodemographic			
Gender			
Male	97.44	2.56	0.01
Female	98.60	1.40	
Age (Years)	44.23±1.01	45.93±065	0.16
Ethnicity			
Mexican	2.07	97.93	0.58
Other Hisp	1.94	98.06	
White	1.91	98.09	
Black	2.07	97.93	
Other Race	2.82	97.18	
PIR	2.87±0.13	3.08±0.05	0.14
PIR<1 (poverty)	12.60	13.05	0.83
Health History			
BMI (kg/m^2^)	30.01±0.49	28.74±0.25	<0.01
Obese	42.19	36.05	0.06
Diabetes			
Yes	1.41	98.59	0.53
No	2.01	97.99	
Phosphorus Intake (mg)	1412.27±57.59	1363.72±23.20	0.35
Steroid Use			
(>last 30 days)	0.86	0.43	0.34
Environmental Phenols			
Urinary			
Benzopheone-3 (ng/mL)			
≤6	98.44	1.56	0.18
>6 & ≤30.1	97.19	2.81	
>30.1	96.70	3.30	
Urinary Bisphenol A (ng/mL)			
<1.3	97.91	2.09	0.61
≥1.3 & <3	97.02	2.98	
≥3	97.04	2.96	
Urinary Tricolsan (ng/mL)			
≤4.2	96.32	3.68	0.18
>4.2 & <23.3	97.68	2.32	
≥23.3	97.97	2.03	

^*^Low hemoglobin is defined as <12 g/dL for women and <13 g/dL for men.

^ Low hematocrit was defined as <36% for women and <41% for men.

**Table 5:** Multivariate logistic regression for phosphorus ≤2.5 mg/dL

**Variable**		**Odds Ratio (OR) 95% CI**	
	**Model 1 (n=13,502)**	** Model 2 (n=4,385)**	**Model 3 (n=4,223)**
Albumin (mg/dL)	0.56 (0.35-0.93)^b^	0.58 (0.21-1.58)	0.89 (0.23-3.44)
BUN (mg/dL)	0.90 (0.86-0.95)^b^	0.86 (0.76-0.98)^b^	0.83 (0.76-0.91)^a^
Uric Acid (mg/dL)	1.04 (0.90-1.21)	1.23 (0.92-1.66)	1.35 (1.09-1.66)^a^
Corr. Calcium (mg/dL)	0.38 (0.23-0.63)^a^	0.77(0.25-2.36)	0.26 (0.07-0.88)^b^
Gender (Female)	0.47 (0.24-0.94)^a^	1.00	0.56 (0.23-1.37)
BMI (kg/m^2^)	1.01 (0.99-1.03)	1.01 (0.95-1.07)	1.00 (0.97-1.04)
Creatinine (ng/mL)	3.19 (0.72-1419)	23.48 (1.10-501.47)^b^	1.17 (0.03-39.53)
Low Ferritin (<12 g/L)		0.14 (0.04-0.45)^b^	
Parathyroid Hormone (pg/ml)			1.01 (0.995-1.03)

^a^p<0.01.

^b^p<0.05.

## Discussion and conclusion

In these cycles of NHANES, there was relatively high percentage of individuals without chronic kidney disease who had hyperphosphatemia (15.29%) in comparison with previous analyses of earlier cycles (NHANES III – 1988–1994) which found 14% in the US population without excluding those with CKD and using a lower threshold ≥ 4.0 mg/dL [39]. The higher percentage may be explained by changes in dietary intakes, particularly an increased consumption of processed foods, with high phosphate contents in the last 10–15 years [40,41].

### Hyperphosphatmeia and anemia

This is the first study to evaluate risk factors for hyperphosphatemia and hypophosphatemia in a large population of US adults without chronic kidney disease. While these results suggest some known factors associated with hyperphosphatemia including an association with increasing calcium levels and female gender and a reduced risk with age, we also found increased risk with anemia, as indicated by low hemoglobin levels, as well as an association with lower BMI. Previous population based studies have found an association between kidney function and anemia in adults with a eGFR < 60 mL/minute but not in adults with normal kidney function [42]. As such, we restricted our study to individuals with a eGFR ≥ 60 mL/minute. The chronic inflammatory state of kidney disease, including higher C-reactive protein levels and lower 25-OH-D levels have been found to be associated with lower hemoglobin levels [43,44]. While our analysis did not include participants with kidney disease, we did not find any association between signs of inflammation including CRP levels, vitamin D and hyperphosphatemia. Kendrick et al. [45] hypothesize that as TNF-alpha interferes with normal erythropoisesis and a decreased response to epoetin, higher vitamin D levels could result in increased hemoglobin through a down-regulation of TNF-alpha and other proinflammatory cytokines.

Additionally, low vitamin D levels decrease gut phosphorus absorption, as vitamin D acts on intestinal epi-thelial cells to increase intestinal absorption of calcium and phosphorus, and hence may be associated with hyperphosphatemia [46]. Additionally, low levels of vitamin D and parathyroid hormone increase the transport of phosphorus out of the renal cells through 3NA-HPO4 co-transporter type 2a (NPT-2a) and limit renal tubular reabsorption additionally resulting in hyperphosphatemia [47,48]. However, under conditions of normal phosphate intake, as was observed with the NHANES population, vitamin D does not stimulate jejunal phosphorus absorption; it is only in patients with chronic kidney disease or low levels of vitamin D, that vitamin D administration can increase phosphorus absorption. Because of the complex relationship between these factors and phosphorus absorption, we adjusted for vitamin D levels in addition to parathyroid hormone in a smaller model examining risk factors for hyperphosphatemia and hypophosphatemia.

In this adjusted model for hyperphosphatemia, the association with low hemoglobin was no longer statistically significant, although it is possible that the smaller model (only one third the size of the larger model) was not sufficiently powered. When the association between low hemoglobin and hyperphosphatemia was assessed stratifying on vitamin D level > 30 ng/ml versus vitamin D level ≤ 30 ng/ml, the odds ratios were not significantly different in between models (OR 1.10 versus OR 1.29) or in comparison with the adjusted model OR 1.33, suggesting that neither confounding nor interaction plays an important role in defining the inter-relationships between serum phosphorus levels, hemoglobin and vitamin D.

Ideally, future cycles of NHANES will include vitamin D and parathyroid hormone levels tested in all participants, as this was one of the limitations of our study, that we were unable to test these associations using one cycle of NHANES.

It is not clear what mechanism is responsible for the increased serum phosphorus levels associated with anemia. Studies of hyperphosphatemia have been almost exclusively in patients with kidney disease, however, given the associations between hyperphosphatemia, cardiovascular disease and increased mortality [49], it is important to better understand the risk factors in the general population, in addition to those with CKD. The mechanisms that underlie the relationship between lower hemoglobin levels and hyperphosphatemia need to be further studies in the general population as they currently are unexplored.

### Hypophosphatemia

We found a low percentage of overall hypophosphatemia in these cycles of NHANES (0.59%). There is limited data on the prevalence of hypophosphatemia in the general population, although approximately 2-20% of hospitalized patients may have hypophosphatemia [50,51], with higher percentages in ICU and critically ill patients.

In contrast to the studies by Haglin [11] and Haglin et al., [52], we did not find any evidence to suggest associations between hypophosphatemia and the metabolic syndrome although increasing BMI neared statistical significance OR 1.01 (0.99-1.03). Haglin et al. found an inverse correlation between serum phosphate and BMI levels in women and an association between higher serum glucose levels and hypophosphatemia in males [11]. Similarly, however, we did find important gender-specific differences in relation to serum phosphorus levels. We found an increased risk in men alone for fasting glucose levels and hypophosphatemia (results not shown). Increased BMI neared statistical significance for association with hypophosphatemia in women controlling for fasting glucose levels (OR 1.06, 95% CI (0.998-1.13) (results not shown). Haglin et al. [52] hypothesize that the mechanism that cause hypophosphatemia in relation to obesity is an overconsumption of a diet with low nutrient density or phosphate depletion due to low protein intake and in animal experiments, hypophosphatemia is associated with glucose uptake [47] and hypophosphatemia is related to reduced glucose tolerance [53]. Lastly, we did not find any association between phosphate levels and steroid use, in contrast with the study by Skversky et al., which found an inverse association between steroid use and vitamin D levels [54].

Future studies need to be longitudinal in nature so as to better understand the mechanisms involved in explaining the relationship between hypophosphatemia and either obesity or serum glucose levels based on gender. Our current understanding is associative in nature and does not evaluate the temporality of relationships.

## Competing interests

The author has no competing interests.

## Pre-publication history

The pre-publication history for this paper can be accessed here:

http://www.biomedcentral.com/1471-2369/14/178/prepub
